# UK Real-World Evidence of Using Durvalumab Plus Cisplatin and Gemcitabine in Advanced Biliary Tract Cancer via an Early Access Scheme

**DOI:** 10.3390/cancers17172732

**Published:** 2025-08-22

**Authors:** Harry Daniels, Mona Hassan, Omer Babiker, William Rowley, Aitzaz Qaisar, Emma Phillips, Ellana Griffin, Catherine Bell, Bahaaeldin Baraka, Shyamika Acharige, Maia Aquino, Rachel Plant, Justin Mencel, Samuel Chan, Dominique Parslow, Arvind Arora, Martin Scott-Brown, Shelize Khakoo, Chiara Braconi, Daniel Palmer, Yuk Ting Ma, Shivan Sivakumar

**Affiliations:** 1 Department of Oncology, Queen Elizabeth Hospital, Mindelsohn Way, Birmingham B15 2GW, UK; omer.babiker@uhb.nhs.uk (O.B.); yukting.ma@uhb.nhs.uk (Y.T.M.); 2 Department of Oncology, Queen Alexandra Hospital, Cosham, Portsmouth PO6 3LY, UK; mona.hassan@porthosp.nhs.uk; 3 The Clatterbridge Cancer Centre NHS Foundation Trust, Clatterbridge Road, Bebington, Wirral CH63 4JY, UK; rowleyw@liv.ac.uk (W.R.); shyamika.acharige@porthosp.nhs.uk (S.A.); samuel.chan@porthosp.nhs.uk (S.C.); palmerd@liverpool.ac.uk (D.P.); 4 Beatson West of Scotland Cancer Centre, Gartnavel General Hospital, 1053 Great Western Rd, Glasgow G12 0YN, UK; aitzaz.qaisar@nhs.scot (A.Q.); chiara.braconi@glasgow.ac.uk (C.B.); 5 Department of Oncology, The Royal Marsden Fulham Road, London SW3 6JJ, UK; emma.phillips@rmh.nhs.uk (E.P.); shelize.khakoo@rmh.nhs.uk (S.K.); 6 Department of Oncology, University Hospital Coventry & Warwickshire, Clifford Bridge Rd., Coventry CV2 2DX, UK; ellanna.griffin@uhcw.nhs.uk (E.G.); catherine.bell@uhcw.nhs.uk (C.B.); martin.scott-brown@uhcw.nhs.uk (M.S.-B.); 7 Deparment of Oncology, City Hospital, Derby Road, Nottingham NG7 2UH, UK; bahaeeldin.baraka@nhs.net (B.B.); arvidarora@nhs.net (A.A.); 8 Department of Oncology, Guy’s Hospital, Great Maze Pond, London SE1 9RT, UK; maia.aquino@gstt.nhs.uk (M.A.); justin.mencel@rmh.nhs.uk (J.M.); 9 Dorset Cancer Centre, Poole Hospital, Longfleet Road, Poole BH15 2JA, UK; rachel.plant@uhd.nhs.uk; 10 Plymouth Hospitals NHS Trust, Plymouth PL6 8DH, UK; dominiqueparslow@nhs.net; 11 Department of Immunology and Immunotherapy, School of Infection, Inflammation and Immunology, College of Medicine and Health, University of Birmingham, Birmingham B15 2TT, UK

**Keywords:** cholangiocarcinoma, durvalumab, immunotherapy, immune checkpoint inhibitors, real world evidence, biliary tract cancer

## Abstract

**Background:** Durvalumab (anti-PD-L1) in combination with gemcitabine and cisplatin has become the first-line treatment for patients with locally advanced, surgically unresectable, or metastatic biliary tract cancer, following the survival benefit demonstrated in the TOPAZ-1 phase III trial. This study presents real-world data from UK centres in patients who received early access to the regimen via AstraZeneca’s scheme. The aim was to assess the safety and efficacy of this treatment approach in routine clinical practice and compare it to outcomes reported in the TOPAZ-1 trial. **Method:** This retrospective study included patients with locally advanced, surgically unresectable, or metastatic biliary tract adenocarcinoma who received durvalumab in combination with gemcitabine and cisplatin. Data were collected across ten UK centres. The primary endpoint was progression-free survival (PFS), with secondary endpoints including overall survival (OS), overall response rate (ORR), and safety outcomes, encompassing both chemotherapy and immunotherapy-related adverse events (AEs). **Results:** A total of 134 patients treated between April 2022 and December 2023 were included. The median follow-up was 12.8 months (95% CI: 11–16.8). The median PFS was 8.83 months (95% CI: 5.73–11.7), closely aligning with the 7.2 months reported in TOPAZ-1 (95% CI: 6.7–7.4). The median OS was 12 months (95% CI: 10.7–13.9), slightly below the 12.8 months observed in TOPAZ-1 (95% CI: 11.1–14.0). The ORR was 29.1% (TOPAZ-1: 26.7%), and the disease control rate was 61.2%. In terms of safety, 64 patients (52.3%) experienced any-grade AEs, and 9 patients (6.8%) had grade 3–4 AEs, representing a lower toxicity profile than TOPAZ-1. Immunotherapy-related AEs occurred in 25 patients (18.7%), with grade 3–4 events in 3%. **Conclusions:** These real-world findings from UK cancer centres support the outcomes of the TOPAZ-1 trial, demonstrating comparable efficacy and a favourable safety profile for durvalumab combined with gemcitabine-cisplatin as first-line treatment for advanced biliary tract cancer.

## Simple Summary

This real-world evidence study assessed the response of patients with locally advanced, surgically unresectable, or metastatic biliary tract cancer who were treated using durvalumab with gemcitabine-cisplatin. The cohort had a median progression-free survival of 8.2 months and an overall survival of 12.7 months. The overall response rate was 29.1% and the disease control rate was 61.2%. Adverse events occurred in 52.3% of patients. The findings align with the TOPAZ-1 trial and other real-world evidence trials and confirm the safety and effectiveness of the treatment regimen in routine clinical analysis.

## 1. Introduction

Biliary tract cancer (BTC), or cholangiocarcinoma, is a heterogeneous disease associated with a dismal prognosis [1]. Early diagnosis followed by resection and adjuvant chemotherapy currently offers the only curative option for patients, though only about 20% of patients are eligible for this. R0 resection confers the best prognosis amongst the patients for whom surgery is appropriate [2], which is achieved in a minority of patients. Most patients with BTC are therefore being treated in the metastatic setting, with this group including locally advanced and metastatic BTC. Historically, there have been limited options available in the palliative setting. The ABC-02 trial, published in 2010, established gemcitabine and cisplatin as the standard of care. Despite being a breakthrough in the management of the disease, the median overall survival from diagnosis was less than a year [3].

Immune checkpoint inhibitors (ICI) targeting the PD1 receptor or the PD-L1 ligand have revolutionized the management of many cancer types, including in the metastatic setting. Phase 1 trials across cancers showed an initial but durable response [4,5]. Initial pre-clinical evidence found BTC tumours were high in PD-L1 expression in the tumour microenvironment [6]. It was suggested that high PD-L1 expression upregulated by inflammatory cytokines in BTC cell lines supported immunoediting.

Preclinical data and early phase trials indicated that PD-L1 inhibitors conferred a survival benefit to patients with BTC [7,8]. This gave the basis for an interventional study to see if the addition of immunotherapy can confer a survival benefit in patients with BTC.

Ultimately, the TOPAZ-1 trial, an international randomized phase 3 trial, established the superiority of using the PD-L1 inhibitor durvalumab in combination with cisplatin/gemcitabine compared to cisplatin/gemcitabine alone [9]. Patients receiving chemotherapy with durvalumab had an estimated 24-month survival of 24.9% (95% CI, 17.9–32.5) when compared with chemotherapy and placebo of 10.4% (95% CI, 4.7–18.8).

Following the success of the TOPAZ-1 trial, durvalumab with cisplatin/gemcitabine was offered to NHS patients via an early access scheme sponsored by the company Astrazeneca, with these being the first cohort of patients to receive this treatment outside of a trial setting. In this study, the data from these patients were analysed to explore the outcomes and confirm the survival benefit observed in TOPAZ-1.

Real-world evidence (RWE) studies are of growing importance in oncology [10], and multiple RWE studies have now assessed the impact of durvalumab with cisplatin/gemcitabine in BTC following TOPAZ-1 [11,12,13] This paper aims to add to that growing body of evidence of the efficacy of ICI therapy in BTC in the UK context and analyse the impact of categorical variables to predict positive response and increased overall survival.

## 2. Method

### 2.1. Study Population

The population of this study included newly diagnosed patients with intrahepatic biliary tract cancer, extrahepatic biliary tract cancer, and gallbladder cancer, which were unresectable, so either locally advanced or metastatic adenocarcinoma, treated with durvalumab with gemcitabine and cisplatin in the first-line palliative setting. Data were obtained from 10 UK cancer centres. The study was approved by the local audit committee at each centre.

Durvalumab was provided by Astrazeneca (Cambridge, UK) via an early access scheme, with this cohort being the first patients to be treated with durvalumab with cisplatin/gemicitabine in the UK prior to NICE approval. Astrazeneca was not involved in the planning, data collection, or data analysis of this study.

A cutoff of 60 U/L was used for an elevated alanine aminotransferase (ALT). A cutoff of 37 U/mL was used for elevated Ca 19.9. A cutoff of 2.5 ug/L was used for elevated carcinoembryonic antigen (CEA).

### 2.2. Statistical Analysis

The primary endpoint of this study was to evaluate progression-free survival (PFS) in months in patients with locally advanced/surgically unresectable/metastatic biliary tract cancer treated with first line palliative durvalumab with cisplatin and gemcitabine in patients treated via the Astrazeneca early access scheme, with PFS defined as the time from initiation of treatment to the time of progression, death, or last follow-up.

Secondary endpoints include evaluating OS (defined as the time in months from initiation of treatment to death or last follow-up), objective response rate (ORR), disease control rate (DCR), safety profile of the treatment in this patient cohort, and the potential impact of categorical variables on the above.

The ORR was assessed by local investigators and is defined as the proportion of patients who achieved either a complete (CR) or partial response (PR), with DCR being the proportion of patients who achieved an ORR and stable disease. Treatment response was evaluated using computed tomography imaging (CT) and categorised by local review.

Adverse events were graded as per CTCAE v5. Survival curves were estimated using the Kaplan–Meier approach. The impact of categorical variables on survival curves in univariate analysis was analysed using log-rank tests. Categorical variables were compared using Fisher’s exact test. A *p*-value of <0.05 was considered statistically significant. Statistical analysis was performed using the Survival package using R version 4.3.1 using packages ‘ggplot’ for figures [14] and ‘Survival’ for statistical analysis [15].

## 3. Results

The data were collected from 134 patients, treated from April 2022 to February 2024, from 10 UK cancer centres. The demographic data on these patients can be found in Table 1.

The cohorts showed some differences in the distribution of BTC subtypes and patient fitness. The average age was 60 years across the cohort. Intrahepatic BTC was the most common subtype, followed by extrahepatic BTC, while gallbladder cancer comprised the smallest group. Notably, patients with intrahepatic disease appeared to be the most fit for systemic therapy, with a higher proportion having a performance status of 0.

Both intrahepatic and extrahepatic biliary tract cancers were more common in men, while gallbladder cancer was more common in women. A higher proportion of extrahepatic and gallbladder cancer patients had undergone prior surgery and were now receiving palliative treatment, whereas most intrahepatic cases were de novo presentations.

There was also variation in the need for drainage procedures before starting treatment, with extrahepatic patients requiring the most interventions. All other clinical characteristics were well balanced across the groups.

**Figure 1:**
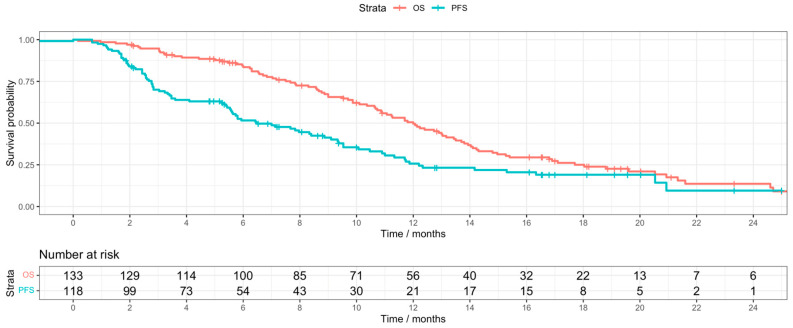
Kaplan–Meier curves for OS and PFS.

As of June 2025, the median follow-up time was 12.8 months (95% CI: 11–16.8 months), with 97 patients (72.4%) having died. A total of 36 patients went on to receive maintenance durvalumab following completion of the treatment regimen, with an average number of 3.5 maintenance cycles across disease sites, with a corresponding interquartile range of 1.75–5 cycles. Across subtypes, the median PFS was 8.33 months (95% CI: 5.73–11.7 months). The median OS was 12 months (95% CI: 10.7–13.9 months) (Figure 1).

When stratifying by disease site, in intrahepatic disease, the median PFS was 5.53 months (95% CI: 2.83–10.1 months), with a median OS of 11.7 months (95% CI: 10.1–13.8 months). In extrahepatic disease, the median PFS was 8.87 months (95% CI: 6.43–12.3 months), with a median OS of 12.4 months (95% CI: 10.3–18.8 months). In gallbladder disease, the median PFS was 5.80 months (95% CI: 3.6–12.2 months), with a median OS of 13.9 months (95% CI: 10.63–13.9 months).

**Figure 2:**
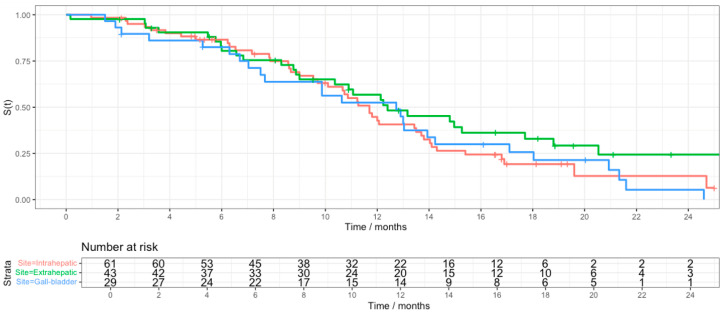
Kapla–Meier curves for OS, stratified by disease site.

There is no overall survival difference between subgroups demonstrated on univariate analysis. At two years, there appears to be a substantial proportion of patients alive with extrahepatic BTC when compared to intrahepatic BTC and gallbladder cancer (24.3% vs. 13% and 5.4%, respectively) (Figure 2).

**Figure 3:**
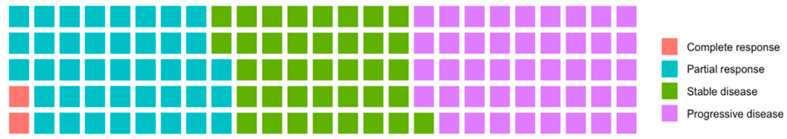
Waffle chart illustrating the response to treatment.

The investigator assessed that the ORR was 29.1% across subtypes, with 39 patients achieving a partial or complete response, with an associated DCR of 61.2% across subtypes (Figure 3). In intrahepatic BTC, the DCR was 50%; in extrahepatic BTC, the DCR was 76.9%; and in gallbladder DCR, the DCR was 65.4%.

A total of 64 patients experienced adverse events (52.3%) of any grade, with 9 patients (6.8%) experiencing a grade 3 or above. A total of 25 patients (18.7%) experienced an immunotherapy-related adverse event, with 4 (3%) patients having a grade 3 or above. A total of 60 (44.7%) patients experienced a chemotherapy-related toxicity of any grade.

In univariate analysis, extrahepatic BTC was associated with an improved DCR when compared to intrahepatic BTC (76.9% vs. 50%, respectively, *p* < 0.05). Disease that was metastatic at treatment initiation was associated with a poorer PFS and OS (*p* < 0.0083). An elevated neutrophil–lymphocyte ratio (NLR) was associated with a poor OS (*p* < 0.05). Of the biochemical markers collected in this cohort, including ALT, CEA, and CA 19.9, no statistically significant difference in OS, ORR, and DCR was demonstrated.

**Figure 4:**
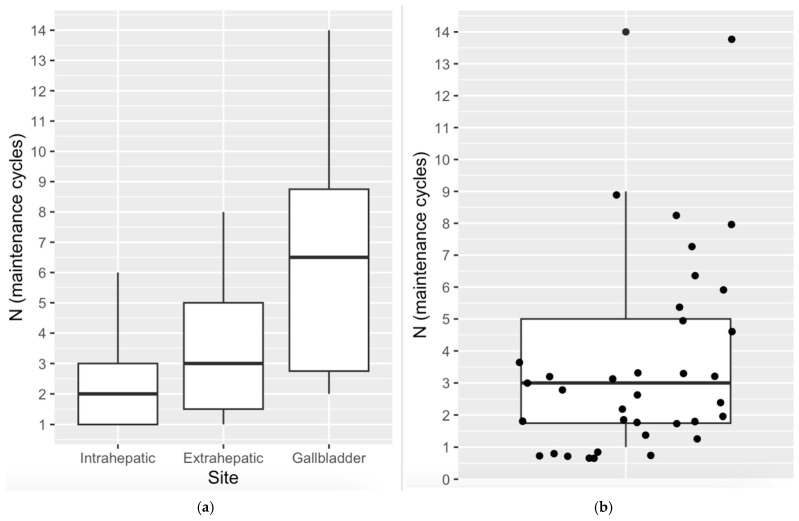
Box plots of patients who received maintenance durvalumab. (**a**) Box plot showing the number of maintenance cycles of durvalumab given to patients stratified by disease site. (**b**) Box plot showing the distribution of the number of cycles of maintenance durvalumab across all disease sites with individual data points.

Of the cohort, 36 patients (28.8%) went on to receive maintenance durvalumab (Figure 4), with patients receiving treatment until disease progression, with an average number of cycles of 3.5. Patients with gallbladder disease received an average of 6.5 cycles, where patients with intrahepatic BTC received 2 cycles, in keeping with the poorer outcomes observed in intrahepatic disease. A summary of the patients who went on to receive maintenance therapy can be found in Table 2. Given the relatively small number of patients who went on to receive maintenance immunotherapy, larger data sets are required to draw robust conclusions on this subset.

## 4. Discussion

Following TOPAZ-1, several multicentre RWE studies have been published in evaluating the durvalumab and gemcitabine/cisplatin in BTC, all of which confirm the results from the TOPAZ-1 trial in the real-world setting. Our results also show that in the UK population, the outcomes with Gemcitabine, Cisplatin, and Durvalumab are similar to those achieved in the Phase 3 TOPA1 trial.

Though not statistically demonstrated at this stage, there appears to be a survival advantage in patients with ECCA relative to ICCA and gallbladder cancer, which cannot be explained by ECCA being understood as a less aggressive disease. In a large population-based study comparing ECCA with ICCA, Liao et al. found that though ECCA had a higher rate of metastasis, there was no statistically significant difference in the OS/cancer-specific survival (CSS) between the two sites [16]. Danese et al. performed multivariate analysis on locally advanced and metastatic BTC and found that ECCA was associated with poorer outcomes, although the cohort included patients not fit for cisplatin/gemcitabine [17]. Further follow-up of this cohort is required to rule out whether this difference is due to cohort size alone.

Contrary to Rimini et al., a statistically significant improvement in DCR/ORR was not demonstrated in patients with an NLR of less than three, across sites or when stratified by disease site. In keeping with the established literature in multiple disease sites [18,19], as well as BTC specifically [20], an elevated NLR was correlated with a reduced survival in this study. In keeping with other RWE studies of durvalumab and cisplatin/gemcitabine use in BTC, no statistically significant difference in OS, DCR, and ORR was demonstrated.

Reduced rates of ADRs compared with the TOPAZ-1 trial can be explained partially by the shorter median follow-up time, and partially by the nature of this study being multicentred, with independent individual physicians reporting ADRs.

As a multicentre RWE study, the PFS of the disease may not be representative. Individual physicians exercised their own independent decisions regarding tumour assessment, and no central review of progression was completed, which may impact the PFS further. Data collection was performed for each site by a local representative, potentially adding bias to our results. The short median follow-up time, particularly in the case of extra-hepatic disease, means these results must be interpreted with caution. Further follow-up of these patients will be conducted, in keeping with other RWE studies of this disease.

## 5. Conclusions

Our results are broadly consistent with TOPAZ-1 and subsequent multicentre RWE studies. There is a benefit to patients for the addition of immunotherapy to chemotherapy in the real-world setting. Further trials are needed to see if this regime can be improved upon.

## Figures and Tables

**Table 1:** Demographic data.

Site	Intrahepatic, N = 62 ^1^	Extrahepatic, N = 43 ^1^	Gallbladder, N = 29 ^1^
Age	65 (59, 71)	67 (60, 73)	61 (55, 72)
ECOG			
0	36 (58%)	15 (35%)	13 (45%)
1	23 (37%)	26 (60%)	16 (55%)
2	3 (4.8%)	1 (2.3%)	0 (0%)
Not available	0 (0%)	1 (2.3%)	0 (0%)
Sex			
Female	21 (34%)	17 (40%)	22 (76%)
Male	41 (66%)	26 (60%)	7 (24%)
Metastatic	45 (76%)	25 (61%)	17 (59%)
Unknown	3	2	0
Previous Surgery	6 (9.8%)	14 (36%)	12 (44%)
Unknown	1	4	2
Drainage/Stent insertion	12 (19%)	25 (58%)	10 (34%)
ALT			
Elevated	14 (23%)	13 (30%)	16 (34%)
Normal range	48 (77%)	30 (70%)	13 (66%)
NLR			
<3	24 (39%)	23 (53%)	16 (55%)
>3	38 (61%)	20 (47%)	13 (45%)
Ca 19.9 at diagnosis			
Elevated	41 (69%)	29 (69%)	19 (70%)
Normal	18 (31%)	13 (31%)	8 (30%)
Unknown	3	1	2
CEA at diagnosis			
Elevated	25 (61%)	21 (66%)	15 (65%)
Normal	16 (39%)	11 (34%)	8 (35%)
Unknown	21	11	6

^1^ Median (IQR); N (%)

**Table 2:** Demographic data on patients who received maintenance durvalumab.

Site	Intrahepatic, N = 16 ^1^	Extrahepatic, N = 17 ^1^	Gallbladder, N = 8 ^1^
Age	66 (60, 71)	67 (58, 72)	62 (60, 66)
ECOG			
0	15 (94%)	6 (35%)	13 (63%)
1	1 (6.3%)	11 (65%)	16 (38%)
Sex			
Female	2 (13%)	8 (47%)	4 (50%)
Male	14 (88%)	9 (53%)	4 (50%)
BMI			
Normal	1 (6.3%)	8 (47%)	2 (25%)
Overweight	9 (56%)	5 (29%)	3 (43%)
Obese	6 (38%)	4 (24%)	3 (38%)
Metastatic	10 (67%)	8 (50%)	4 (50%)
Unknown	1	1	0
Previous Surgery	2 (13%)	6 (40%)	3 (43%)
ALT			
Elevated	3 (56%)	5 (29%)	4 (25%)
Normal range	13 (81%)	12 (71%)	6 (75%)
NLR			
<3	9 (56%)	10 (59%)	4 (50%)
>3	7 (44%)	7 (41%)	4 (50%)
Ca 19.9 at diagnosis			
Elevated	9 (56%)	12 (71%)	3 (38%)
Normal	18 (31%)	13 (31%)	8 (30%)
Unknown	3	1	2

^1^ Median (IQR); N (%).

## Data Availability

This study contains confidential patient data in the context of an audit, so they are not available for the research community.
